# Social Listening in Eastern and Southern Africa, a UNICEF Risk Communication and Community Engagement Strategy to Address the COVID-19 Infodemic

**DOI:** 10.1089/hs.2020.0226

**Published:** 2021-02-18

**Authors:** Silvia Sommariva, Jenna Mote, Helena Ballester Bon, Herisoa Razafindraibe, Domoina Ratovozanany, Vanou Rasoamanana, Surangani Abeyesekera, Parvina Muhamedkhojaeva, Tasmia Bashar, John James, Massimiliano Sani

**Affiliations:** Silvia Sommariva, MPA, MSc, and Jenna Mote, MPH, are Consultants, Communication for Development (C4D), United Nations Children's Fund (UNICEF) Eastern and Southern Africa Regional Office, New York, NY. Helena Ballester Bon, MA, and Massimiliano Sani, MA, are Specialists, C4D, UNICEF Eastern and Southern Africa Regional Office, Nairobi, Kenya. Herisoa Razafindraibe, MRes, is a Specialist and Domoina Ratovozanany, MA, is an Officer, C4D, UNICEF Madagascar Country Office, Antananarivo, Madagascar. Vanou Rasoamanana, MA, is a Specialist, C4D, UNICEF Comoros Country Office, Moroni, Comoros. Surangani Abeyesekera, PG, is a Manager, C4D, UNICEF Kenya Country Office, Nairobi, Kenya. Parvina Muhamedkhojaeva, PhD, is a Manager, C4D, UNICEF Malawi Country Office, Lilongwe, Malawi. Tasmia Bashar, MSc, MA, is a Specialist, C4D; and John James, MSc, is Chief, Communications; both in the UNICEF Zambia Country Office, Lusaka, Zambia.

**Keywords:** COVID-19, Infodemic, Risk communication, Community engagement, Social and behavior change communication, Public health preparedness/response

## Abstract

The coronavirus disease 2019 (COVID-19) pandemic has been closely tied with what has been called an infodemic, a “second disease” that occurs when massive information volumes (particularly with a high prevalence of false information) hinder the public health response. In this context, social listening, the process of monitoring and analyzing conversations to inform strategic activities both online and offline, becomes an even more essential component of risk communication and engagement strategies. In the Eastern and Southern Africa region, the United Nations Children's Fund (UNICEF) and partners in the response have activated their capacity to gather insights on the information needs of the populations served to better inform and engage with local communities. We describe the social listening approach taken at the Eastern and Southern Africa regional level to respond to COVID-19 and highlight efforts by the Comoros, Kenya, Madagascar, Malawi, and Zambia UNICEF country offices to implement digital and nondigital social listening to inform risk communication and community engagement. The analysis highlights channels leveraged, types of data monitored, and provides examples of social listening data use, as well as early challenges and lessons learned.

## Background

Children's lives have been highly, and often silently, affected by the coronavirus disease 2019 (COVID-19) pandemic. Access to lifesaving immunization services, education, protection, mental health, and nutrition are just some of the areas that have been adversely impacted by the crisis.^1^ COVID-19 has exacerbated child poverty, with data showing an estimated 15% increase in the number of children living in deprivation since the start of the pandemic.^2^ In the Eastern and Southern Africa region, a 25% increase in the number of children suffering from acute malnutrition was estimated in 2020 compared with the prior year.^3^ These adverse impacts of the crisis as well as the immediate need to contain disease spread have called for a multilevel and collaborative response effort both globally and locally.

Risk communication and community engagement (RCCE) is a component of crisis response that requires a collaborative and evidence-based approach.^4,5^ In the context of the COVID-19 pandemic, risk communication is the real-time exchange of information, opinions, and advice between response teams and families who are faced with the pandemic threat,^6^ and community engagement is a mutual partnership between responders and communities in affected areas, whereby local stakeholders have ownership in controlling the spread of the outbreak.^7^ The engagement component of RCCE is one of the primary approaches to social and behavior change.^8^ During the pandemic, particularly in a context where there have not been widely available vaccines or therapeutics, behavior change has been central to mitigating COVID-19. The characterization of the COVID-19 pandemic as an infodemic (an overabundance of information—both accurate and not—that undermines the response efforts),^9^ in addition to the epidemiological component of the crisis, has led institutions working on the response to further refine RCCE strategic plans and guidance to meet the challenge of a harmful overflow of information that can hinder risk perception and affect individual health decision making.

Since the early stages of the pandemic, Eastern and Southern Africa region RCCE activities—collaboratively carried out by the United Nations Children's Fund (UNICEF), International Federation of Red Cross and Red Crescent Societies (IFRC), World Health Organization (WHO), and RCCE partners—have been informed by the collection and review of social data. These social data are gathered through a variety of channels including interactive radio, hotlines, short message service (SMS) messages, face-to-face social mobilization, community meetings, social media, messaging apps, and chat bots. For example, the Eastern and Southern Africa region COVID-19 RCCE Interagency Working Group has developed a framework to code, analyze, and triangulate qualitative sociobehavioral data to inform the response to the pandemic.^10^ Insights from social data have been instrumental to ensuring that COVID-19 communication for behavior change and program delivery are aligned with concerns and needs expressed by the communities served.

Several mechanisms have contributed to the evidence base needed to engage with communities about COVID-19 risks. One approach to gather insights that has become key to behavioral response strategies is social listening,^8^ meaning the tracking, analysis, and synthesis of community inputs both digital and offline. Social listening identifies questions and queries, as well as concerns, complaints, and suggestions shared by communities. This approach can help identify rumors—information that has not been verified—and false information (misinformation and disinformation).^11^ Data from social listening in the context of the COVID-19 pandemic, triangulated with other sources of insights such as primary research data, can contribute to social and behavioral sciences evidence, which in turn provides a holistic understanding of the dynamics of disease outbreak and a more effective response.

The digital dimension of social listening has emerged as highly relevant to the COVID-19 pandemic, complementing the already established interagency work on community feedback data in the region led by IFRC. Online platforms, including social media and instant messaging apps, have been a key vehicle for spreading false information about COVID-19 and fueling the infodemic.^12,13^ In this context, organizations involved in the region's response have invested in integrating insights from digital platforms into existing strong mechanisms for gathering community feedback on the ground. The intent to monitor COVID-19-related conversations online is to complement listening data from offline mechanisms to understand concerns and questions and manage the information disorder.^14^

This article describes key elements of a social listening strategy, based on the experience of UNICEF in the Eastern and Southern Africa region and in 5 countries within the region. In particular, the UNICEF country office examples show how elements of digital and offline social listening built collaboratively by partners blend together to inform RCCE activities at the national level.

## Key Elements of a Social Listening Strategy

### Stated Objectives and Scope

Clear objectives and a defined scope of work help circumscribe social listening activities to identify data points that can inform decision making within national responses. Because social listening is a resource-intense activity, often carried out with limited resources and time constraints due to a rapidly evolving social and media context, it needs to be situated within the established RCCE goals.

The Eastern and Southern Africa Regional Social Media Monitoring and Rumor Management Strategy to Support RCCE on COVID-19, developed in June 2020 by the UNICEF Eastern and Southern Africa Regional Office (ESARO), aimed to contribute to 3 objectives related to the COVID-19 RCCE: (1) mitigate potential effects of misinformation on adoption of recommended behaviors, demand, and uptake of services by implementing a system to track, analyze, and manage COVID-19-related rumors at the regional level; (2) inform the design and implementation of high-quality digital communication for content that meets information needs and responds to concerns and rumors shared by different audiences; and (3) reinforce country-level capacity in rumor tracking and management by providing guidance to country offices on social media monitoring activities applied to the COVID-19 response.

### Established Framework

Any social listening exercise is embedded within the larger communication ecosystem it is trying to understand and feed into. Information on rumors, questions, or concerns expressed through existing media platforms is integrated, categorized, and analyzed to produce actionable insights. This process ultimately informs risk communication and engagement strategies to fight the COVID-19 pandemic, through both digital and nondigital media. The underlying approach relies on the concepts of integration between online and offline channels as well as horizontal collaboration with RCCE partners working on the response in the region. As the most vulnerable populations are more likely to be disconnected from digital platforms, consideration of alternate tools is necessary to avoid exacerbating inequities or deepening the digital gap.

**Figure 1.**
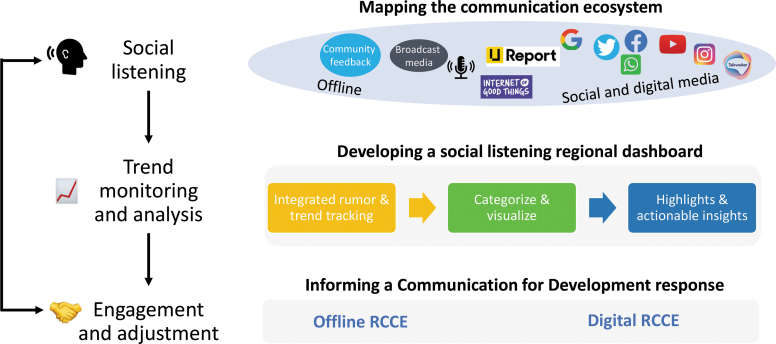
UNICEF Eastern and Southern Africa Regional Office social listening strategy. Abbreviation: RCCE, risk communication and community engagement.

Figure 1 provides an overview of the social listening regional framework adopted by UNICEF ESARO, which includes social listening, trend monitoring and analysis, and engagement and adjustment. This framework aligns with the work conducted by other organizations working on the COVID-19 response in the region. For example, WHO developed an infodemic management framework in April 2020, after consultations with more than 1,300 experts from different fields. That framework is based on 4 main pillars: identify information gaps and misinformation, simplify the technical knowledge, amplify the correct information and debunk misinformation, and quantify the impact (of the infodemic and resulting interventions).^15^ The WHO framework has been adopted by the Africa Infodemic Response Alliance, a WHO-hosted network of intergovernmental, governmental, and nonstate actors that was launched in December 2020 to coordinate and maximize efforts in combating misinformation around the COVID-19 pandemic and other health emergencies in Africa, with a strong focus on digital channels.^16^

### Resource Mapping

A high-level mapping of the regional communication ecosystem and tools already available to monitor its components helps maximize the local relevance of social listening activities and enhancement of existing platforms (Table 1). These include mechanisms to track feedback offline (eg, using phone-administered polls, national COVID-19 hotlines, or in-person community feedback) and through broadcast media monitoring, digital media monitoring, mobile engagement initiatives, and analysis of data from social media platforms.

**Table 1.** Summary of Social Listening Activities

Country	Channels for Social Listening	Type of Data Tracked via Digital Social Listening Monitored
Kenya	Online/social media monitoring, Internet of Good Things, hotline, SMS, radio	• Information on the content of specific COVID-19 rumors or myths shared in the country
		• Metrics of reach or engagement of COVID-19 rumors or myths on social media in the country
		• Questions or search trends related to COVID-19 shared in the country
		• Most popular COVID-19 social media posts or news articles in the country
Comoros	Online/social media monitoring, hotline, village community leaders	• Information on the content of specific COVID-19 rumors or myths shared in the country
Madagascar	Online/social media monitoring, local community leaders, radio, TV, news media	• Information on the content of specific COVID-19 rumors or myths shared in the country
		• Metrics of reach or engagement of COVID-19 rumors or myths on social media in the country
		• Questions or search trends related to COVID-19 shared in the country
		• Most popular COVID-19 social media posts or news articles in the country
Malawi	Online social media monitoring, U-Report, Internet of Good Things	• Questions or search trends related to COVID-19 shared in the country
Zambia	Online/social media monitoring, U-Report, news media, radio, social mobilizers	• Information on the content of specific COVID-19 rumors or myths shared in the country
		• Questions or search trends related to COVID-19 shared in the country
		• Most popular COVID-19 social media posts or news articles in the country

Abbreviations: COVID-19, coronavirus disease 2019; SMS, short message service.

### Standards for Listening, Analysis, and Dissemination

Establishing a routine for social listening activities—which include monitoring channels, reviewing the portfolio of tools used to conduct the monitoring, and writing keyword search strings—helps to ensure the consistency of data collection over time and to maximize the ability to pick up early signals of misinformation and rumors. For digital conversations, metrics tracked typically focus on volume, reach, and engagement. The use of shared taxonomies in structured rumor logs helps identify the main topics of interest in the conversations tracked and adds qualitative insight. For example, in an effort to harmonize and compare findings, partners within Africa Infodemic Response Alliance have combined internal bespoke taxonomies with the use of the WHO Infodemic Management 4 thematic areas—cause, characteristics of the illness, treatment, and interventions^17^—as a shared framework of analysis.

Finding ways to sustainably disseminate insights is also necessary. For example, UNICEF ESARO has adopted a dissemination model that combines periodic reporting of social listening insights on COVID-19 with the use of interactive dashboards to display data on rumors and trends or questions, made accessible to all country offices and response partners. Dissemination at the country level with national partners and stakeholders in the response is done by country offices through existing RCCE mechanisms.

### Monitoring Activities

Defining key indicators and setting up structures to monitor ongoing performance are challenging and important aspects of a social listening strategy. As with objectives, selected indicators should be aligned with broader monitoring and evaluation frameworks.

For example, ongoing digital listening activities conducted by UNICEF ESARO have been providing context for UNICEF RCCE indicators such as “number of people sharing their concerns and asking questions/clarifications for available support services to address their needs through established feedback mechanisms” (COVID-19 Humanitarian Action for Children [HAC] indicator 3),^18^ “number of people who participate in COVID-19 engagement actions” (where engagements are defined to include “active dialogue to respond to rumors and misinformation”) (RCCE indicator 2),^18^ and the process indicator “number of rumor tracking reports” outlined in the UNICEF ESARO monitoring and evaluation framework to assess progress of the implementation of the COVID-19 RCCE response.^19^ Between May and December 2020, digital social listening conducted by UNICEF ESARO has tracked over 2,500 rumors, questions, or concerns about COVID-19 expressed by online users through social media or digital news comment spaces. Misinformation tracked has generated at least half a million engagements/interactions on social media. Insights have been disseminated through biweekly and monthly reports (20 weekly/biweekly reports and 8 monthly reports between May and December 2020).

### Capacity Building and Engagement

**Figure 2.**
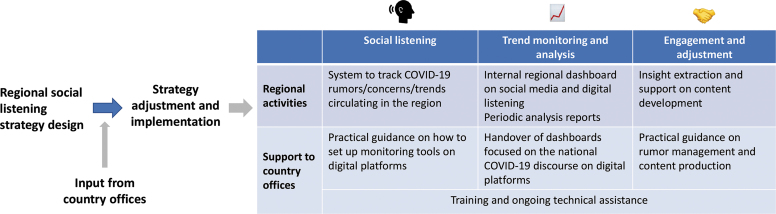
Regional strategy implementation.

Sustainability of a social listening strategy requires enhancement of response partners' social listening and infodemic management capacity. Several workstreams are ongoing in the region in this respect. For example, the Africa Infodemic Response Alliance provides technical assistance to countries in tracking and managing rumors related to COVID-19, vaccines, and polio, while supporting the creation of a workflow that would implement the 4 pillars of the WHO infodemic management framework. UNICEF ESARO has developed several practical tools to reinforce social listening at country office level in the Eastern and Southern Africa region, including a series of practical guides on digital monitoring tools, insight extraction, and rumor management, as well as the development of tailored digital monitoring dashboards for use by national partners and country offices. Figure 2 provides an overview of how the implementation process is unfolding as the infodemic continues to affect the pandemic response.

Insights from social listening data have also been used to engage with key partners in the region, particularly religious leaders and communities. Under the global Faith in Action COVID-19 Initiative, which aims to mobilize faith leaders on key actions including misinformation and rumor management, UNICEF ESARO—in partnership with the African Council of Religious Leaders and others—has worked collaboratively to build misinformation literacy and social listening capacity, highlighting the unique role played by religious leaders in influencing behaviors and beliefs that affect families' and children's wellbeing.

## Social Listening Activities in National COVID-19 Response

### Comoros

To collect feedback offline, the UNICEF Comoros country office partnered with a mobile company and the National Regulation Authority of Information and Communications Technology to establish a free 24 hours a day/7 days a week COVID-19 hotline (1717), with 3 call centers and 54 call operators on the 3 islands of Ngazidja, Ndzouani, and Mwali. The 1717 hotline was the first tool for interaction with communities to collect feedback, disseminate information, and refer suspected cases of COVID-19, and the hotline operators collect information on circulating rumors and misconceptions shared by callers. Feedback is also collected through the work of over 400 village community leaders who submit reports on perceptions, concerns, and rumors related to COVID-19. The UNICEF Comoros country office and national partners monitor online conversations through the digital monitoring dashboard, including UNICEF's mentions on social and digital news media. Online and offline monitoring are bridged by holding regular meetings with the RCCE national committee, where insights from all available social listening data sources are presented. Members participate in regular information sharing via a WhatsApp group, which has proved particularly important at key moments of the COVID-19 outbreak to monitor rumors and misinformation and to decide if and how to respond. Insights shared in the RCCE subgroup are used to adapt the RCCE COVID-19 strategy and activities.

### Kenya

UNICEF Kenya has partnered with Safaricom, a communications company in Kenya, to establish a national call center to provide information on COVID-19. In addition to the use of an interactive voice recorded mechanism with a bot named Zuri (which operates like a chat function), 50 telephone operators track misinformation or rumors raised by callers. A partnership has also been developed with Africa's Voices Foundation, a platform using radio and SMS to enable 2-way communication and track trends and misinformation that arise from questions discussed on live radio shows and through SMS interactions.

The Internet of Good Things^20^—a UNICEF-led initiative to bridge the digital divide and increase societal knowledge—is used to administer online polls on information gaps and risk perception on a rolling basis. It has also been used to conduct a quiz to collect information on COVID-19 understanding and misconceptions while reinforcing accurate information. This is complemented by tracking the content of specific COVID-19 rumors or myths shared in the country, metrics of reach or engagement of COVID-19 rumors or myths on social media, questions or search trends related to COVID-19, and the most popular COVID-19 social media posts or news articles in the country through the digital monitoring dashboard.

UNICEF Kenya also leverages data shared by external partners to complement social listening activities, particularly to ensure the voices of young people are included in the conversation. For example, through a partnership with Shujaaz Inc. (“heroes”), an interactive multimedia youth platform that promotes interactive engagement on COVID-19-related issues with adolescents. Shujaaz consolidates and shares highlights from these conversations through reports such as the Shujaaz Barometer.

Through a specific social listening subgroup, key highlights and actionable insights from both online and offline social listening are disseminated and discussed, both internally and externally, to the national multistakeholder RCCE coordination group. A messaging matrix that all actors use to produce communications materials is updated as the situation evolves and as behavioral insights and necessary responses to social listening and feedback emerge.

### Madagascar

Jointly with the Ministry of Public Health and other partners, UNICEF Madagascar is supporting an emergency hotline (910, referred to as the “green line”) developed in 2017 following high-level advocacy to enable people to ask questions and provide feedback during outbreaks and emergencies, including now to support the COVID-19 response. To complement this at the local level, community leaders and agents collect feedback and questions from their regular interactions with community members, and local public health departments relay the collected information twice each month. Data collected are analyzed at the central level by the Information Watch and Rumor Mitigation working group within the National Communication Sub-Committee for the Fight Against Epidemics. Online, UNICEF conducts periodic reporting on questions, search trends, content, and metrics on reach or engagement of specific COVID-19 rumors, myths, or popular posts shared on social media in the country highlighted via the digital monitoring dashboard. The Ministry of Public Health is supported at central level to conduct daily information monitoring of news and social media related to COVID-19 in collaboration with a communications agency, which tracks rumors and sensitive information through several channels including radio, social networks, TV, newspapers, and the 910 hotline.

To disseminate and discuss key insights, the Communication Sub-Committee For the Fight Against Epidemics organizes a monthly national virtual communication workshop with all the communication and health promotion focal points from the country's 22 administrative regions. The subcommittee reviews the data and updates the communication plan, for example, through a bank of key messages that is regularly updated based on key findings from social listening. The national RCCE plan also has been adjusted and reoriented in light of social listening evidence collected.

### Malawi

UNICEF Malawi conducts online and social media monitoring (using the tailored digital monitoring dashboard), along with leveraging UNICEF-owned channels such as U-Report and the Internet of Good Things to launch mythbuster quizzes and collect data on public understanding, opinions, trust, and confidence, while illuminating information on questions, rumors, and potential hesitancy. U-Report polls have collected feedback on distance learning and returning to school; violence against children and gender-based violence; access to services; knowledge, attitudes, and practices related to COVID-19; and more. Similarly, polling on the Internet of Good Things has collected feedback on wearing face masks and feelings and experiences during the COVID-19 pandemic, particularly from target audiences such as students.

### Zambia

UNICEF Zambia supported the establishment of a short-term COVID-19-specific call center for the public to report concerns and receive information on the COVID-19. Online, UNICEF Zambia administers mythbuster polls through U-Report and engages users with an SMS bot. The SMS bot provides a menu from which users can access various COVID-19-related information categories. Users can engage in direct 2-way SMS interaction with the bot, which responds to questions on news spreading internationally or on some local media or social media platforms. UNICEF, with the input of partners, develops a weekly social listening report that analyzes social listening insights from a review of news, WhatsApp groups, social media, and U-Report. The report is shared with government chairs and through a mailing list that includes those involved in public messaging and advocacy related to COVID-19 and the subcommittee members. In the future, weekly reporting will include insights from a call center and the Internet of Good Things. The national RCCE subcommittee that works on dynamic listening and rumor management uses the weekly social listening report to monitor the conversation on COVID-19 happening in the country to produce messaging that adapts to shifting myths and information needs.

## Challenges and Lessons Learned

The country examples provide insights into how elements of a social listening strategy are translated into collaborative response at the national level. To this end, while the infodemic is in large part fueled by information sharing on digital platforms, efforts in triangulating insights from digital social listening with other sources of data emerge clearly. However, online and offline social listening mechanisms are set up differently in terms of governance structures both internally and externally, which creates challenges in harmonizing the reporting and triangulation of insights.

The speed at which the COVID-19 pandemic and its associated infodemic spread has also required social listening systems to be set up quickly with available tools. At the regional level, use of commercially available tools for social media and digital monitoring has been preferred to ensure feasibility and quick update of monitoring and reporting activities during the crisis. While these tools allow for some degree of customization, they are usually built for brand monitoring and business development. In the long term, it will be important to consider the development of ad hoc monitoring platforms for digital monitoring that can easily integrate with existing channels of feedback (eg, U-Report or Internet of Good Things) and focus on the needs of the communities served.

Finally, measuring the use of social listening data and their impact both upstream and downstream is also an area where further work is needed. An internal survey administered by UNICEF ESARO to country offices (N = 17) in July and August 2020 found that most country offices have been using information from digital social listening activities to share with national partners or internally to support decision making on COVID-19 RCCE and the broader response (n = 17; 100%), create content to address questions posed by users online (n = 11; 65%), make decisions on when to promote certain pieces of content based on trends tracked (n = 11; 65%), and to a lesser extent, directly debunk popular rumors (n = 6; 35%).

The survey findings are in line with the uses of social listening data reported in the country examples presented. For example, in Zambia, communication strategies have been adapted to respond to behavioral insights identified through social listening, including the ways in which stigma could be causing some to hide when they have COVID-19 symptoms, community resistance to sensitization efforts due to expectations of a distribution of face masks, soap, or hand sanitizer, and some fear of going to health facilities for routine services. In Kenya, insights from social listening have prompted key shifts in messaging toward focusing on social obligation and social trust. In Comoros, the RCCE COVID-19 strategy and activities have been adapted to focus more on rebuilding trust between the Comorian population, health workers, and leaders in the COVID-19 response due to social listening insights highlighting distrust.

In addition to these output indicators and anecdotal evidence of how social listening data influence decision making among national response partners, expanding our understanding of the impact of this work on communities is key. In many countries in the region, social listening capacity needs to be strengthened to achieve set targets for indicators on the number of people sharing concerns and asking questions for available support services to address their needs through established feedback mechanisms. Tracking the impact of these activities on health and other outcomes for children and families is needed to reinforce or adjust ongoing activities and for programmatic planning.

## Conclusions

The COVID-19 pandemic has highlighted the importance of leveraging and reinforcing regional- and country-level social listening capacity, particularly on digital platforms. Moving forward, resources built with a specific focus on the immediate COVID-19 response are being adapted and expanded to meet ongoing challenges such as the social impact of the pandemic on access to services and to prepare for COVID-19 vaccine rollout. The continuation and strengthening of these mechanisms are key to emergency preparedness and response plans beyond COVID-19 to allow for proactive management of future infodemics.
